# Optimizing the utilization of aluminum adjuvants in vaccines: you might just get what you want

**DOI:** 10.1038/s41541-018-0089-x

**Published:** 2018-10-10

**Authors:** Harm HogenEsch, Derek T. O’Hagan, Christopher B. Fox

**Affiliations:** 1 Department of Comparative Pathobiology, College of Veterinary Medicine, Purdue University, West Lafayette, IN USA; 2 Purdue Institute of Inflammation, Immunology and Infectious Diseases, Purdue University, West Lafayette, IN USA; 3 GSK Vaccines, Rockville, MD USA; 4 IDRI, Seattle, WA USA; 5 Department of Global Health, University of Washington, Seattle, WA USA

## Abstract

Aluminum-containing adjuvants have been used for over 90 years to enhance the immune response to vaccines. Recent work has significantly advanced our understanding of the physical, chemical, and biological properties of these adjuvants, offering key insights on underlying mechanisms. Given the long-term success of aluminum adjuvants, we believe that they should continue to represent the “gold standard” against which all new adjuvants should be compared. New vaccine candidates that require adjuvants to induce a protective immune responses should first be evaluated with aluminum adjuvants before other more experimental approaches are considered, since use of established adjuvants would facilitate both clinical development and the regulatory pathway. However, the continued use of aluminum adjuvants requires an appreciation of their complexities, in combination with access to the necessary expertise to optimize vaccine formulations. In this article, we will review the properties of aluminum adjuvants and highlight those elements that are critical to optimize vaccine performance. We will discuss how other components (excipients, TLR ligands, etc.) can affect the interaction between adjuvants and antigens, and impact the potency of vaccines. This review provides a resource and guide, which will ultimately contribute to the successful development of newer, more effective and safer vaccines.

## Introduction

Vaccination is one of the major contributors to the global control of infectious diseases in the human population. It has been estimated that vaccination has prevented more than 100 million cases of infectious diseases in people since the 1920s in the United States alone.^1^ In recent years, advances in biotechnology, molecular biology, and immunology have led to an increasingly rapid identification of new vaccine antigens and the generation of more effective and safer vaccines. Since most highly purified recombinant antigens are poorly immunogenic, adjuvants are often required to increase the level and duration of protection induced by vaccines. The immune enhancement effect of precipitation of diphtheria toxoid with insoluble aluminum salts was first reported by Glenny et al. in 1926.^2^ Since that time, aluminum-containing adjuvants have been incorporated into billions of doses of vaccines and administered annually to millions of people. This success can be attributed to the fact that aluminum adjuvants are effective with many of the various vaccine antigens in currently licensed vaccines; have an excellent safety profile; are associated with minimal reactogenicity; and are inexpensive.^3,4^ Moreover, they have a practical advantage by offering the possibility of creating single liquid vials or prefilled syringe formats, which are generally preferred in the market place.

In recent years, oil-in-water emulsions, such as MF59 and ASO3, and liposomes have also been included as adjuvants in certain approved human vaccines, and a much larger number of candidate adjuvants are in various stages of discovery and development.^5,6^ Nevertheless, a key message that we would like to deliver is that during the early phases of discovery and development of new adjuvants, we believe that it is necessary that the new approaches should be evaluated competitively with the more established aluminum adjuvants, to determine if the new approach offers a genuine advantage. In addition, to give the aluminum adjuvants a chance to perform optimally, it is necessary that the required time and effort are taken to create a high-quality formulation, with which to make the comparison. To offer some necessary criticism to the field, we are collectively disappointed when a new adjuvant approach is presented in a presentation or a publication, without appropriate “benchmarking” through comparison with the more established aluminum adjuvant, to allow an accurate determination of the level of performance of the new approach. Unfortunately, even when aluminum adjuvants are included in experimental studies, it is not clear that the background work has been done to optimize the aluminum adjuvant, thus not allowing an accurate comparison, on a “level playing field”. We do not believe that the science of adjuvants is well served unless the field collectively grasps the need for appropriate benchmarks in all studies, and that is a key message we would like to deliver in this article. Overall, given the firmly established record of safety and efficacy, we believe that aluminum adjuvants should be considered as the “gold standard” against which all new adjuvant candidates are compared.

**Fig. 1:**
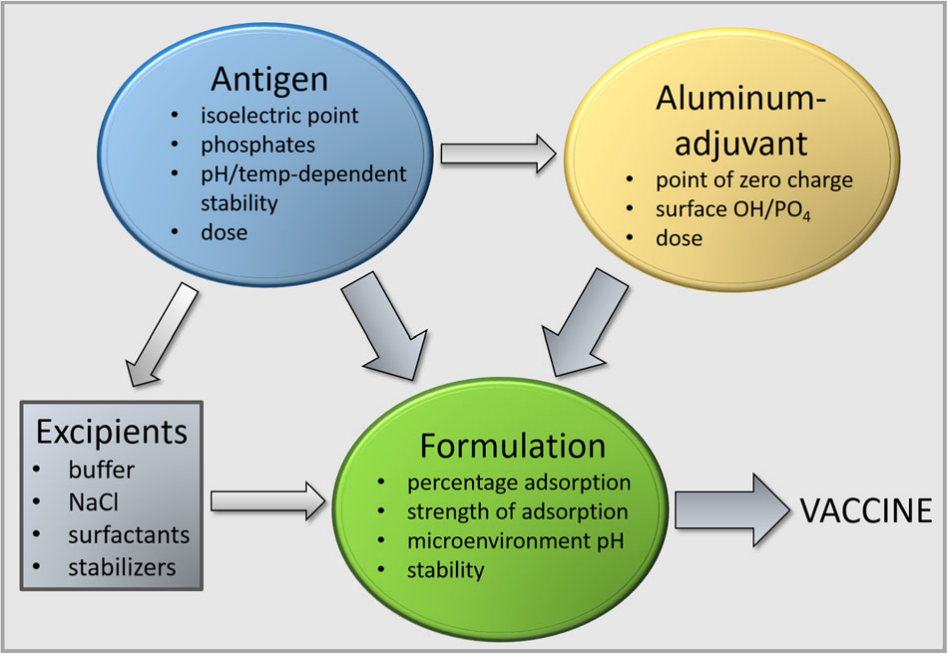
Formulation of an effective vaccine with aluminum adjuvants requires knowledge of the chemical and physical properties of the adjuvant and rigorous characterization of the antigen and antigen−adjuvant formulation. Excipients can affect the antigen−adjuvant interactions

During the early stages of the development of a potential new vaccine, the need for adjuvants should be critically assessed. If it is concluded that an adjuvant is required to obtain a protective immune response, then the vaccine antigens should be evaluated in combination with aluminum adjuvants, before more potent adjuvants are assessed. If the widely available aluminum adjuvants are deemed sufficient, then there is no need to evaluate more potent adjuvants, although clinical trials may be necessary to determine this more accurately. Moreover, the evaluation of aluminum adjuvants requires a serious effort to develop a “stage appropriate”, stable and effective vaccine formulation. The creation of appropriate formulations with aluminum adjuvants requires detailed knowledge of the physical and chemical properties of the vaccine antigen(s), the interaction of antigens with the adjuvant, and knowledge of the effect of excipients such as salts, buffers, and tonicity modifiers (Fig. 1). Lack of attention to these features will likely result in an inadequate formulation and a suboptimal immune response to the vaccine. In addition, the aluminum-adjuvanted comparator, once established, should be carried forward in subsequent studies, so that the enhanced performance of the new adjuvant can be benchmarked consistently across a number of studies with diverse readouts. This will allow a clear definition of how and where the new adjuvant offers improvements over the more established approach, which can be used to support the clinical development plans.

In spite of the widespread and longstanding use of aluminum adjuvants, a more thorough understanding of their physical chemistry, and the necessary formulation steps to allow their optimal use has only begun to emerge more recently.^7,8^ Here, we review current knowledge and provide practical insights into the formulation of vaccines with aluminum adjuvants, we highlight recently developed techniques to assess the integrity and stability of adsorbed antigens, and identify gaps in our knowledge that currently limit the rational design of aluminum-adjuvanted vaccines. However, rather than a comprehensive review of how to create optimal vaccine candidates using aluminum adjuvants, we intend this review as an overview, although we will highlight where to go to find the necessary information to take the next steps, some of which has been reviewed previously.^9–11^

A key message we would like to deliver is that there is a lot of science around the use of aluminum-based adjuvants that is too often underappreciated, or even ignored. Too often aluminum adjuvants appear to be considered as a “fixed” material to which antigens can be simply added (mix and inject approach), while we contend that this is not the case. There are many considerations that need to be brought into play when considering how to evaluate aluminum salts as an adjuvant, which could result in a significantly improved performance. Unfortunately, too often an aluminum adjuvant is used without optimization, resulting in it being dismissed too readily in favor of newer, more exploratory, approaches, with uncertain paths to development. Hence, we will attempt to offer clarity on why formulations with aluminum adjuvants need to be optimized to get a clearer picture of potential performance, while highlighting in broad terms how this might be accomplished.

## Aluminum adjuvants

The first aluminum-adjuvanted vaccines were prepared by the addition of base to a solution of antigen mixed with aluminum potassium sulfate, resulting in precipitation of the antigen and aluminum salt.^2^ Although the term alum is used colloquially to refer to all aluminum adjuvants, it is technically a misnomer since alum is the chemical common name for a solution of aluminum potassium sulfate. More importantly, the term alum does not specify the type of aluminum adjuvant that is used in a vaccine, which has important implications for vaccine formulations. Preparation of vaccines by precipitation of antigens with alum has now largely been replaced by adsorption of antigens to preformed aluminum gels, because precipitation does not give reproducible results and does not allow control over the degree of adsorption.^12^ The two types of aluminum adjuvants commonly used in licensed vaccines are aluminum hydroxide adjuvant (AH) and aluminum phosphate adjuvant (AP). These adjuvants are prepared in house by vaccine companies or purchased from manufacturers such as Brenntag Biosector, Chemtrade, and SPI Pharma^TM^. They are sometimes referred to by their tradenames, such as Alhydrogel®, Rehydragel^TM^, and Adju-Phos®. Different sources of AH and AP are likely to differ in their physicochemical and biological properties, but we are not aware of any reports of direct comparisons that are available in the peer-reviewed literature.

AH is chemically crystalline aluminum oxyhydroxide, AlOOH. It is prepared by mixing an aluminum solution, usually AlCl_3_ or alum [AlK(SO_4_)_2_], with sodium hydroxide. The suspension is then dehydrated under hydrothermal conditions.^13,14^ The degree of crystallinity of the salts depends on the manufacturing conditions, and affects the adsorptive capacity and the speed of dissolution in vitro and in vivo. Poorly crystalline AH with very small crystals as determined by X-ray diffraction has a larger surface area and larger adsorptive capacity than AH with a higher degree of crystallinity.^13–15^ The aluminum at the surface of AH is coordinated with amphoteric hydroxyls that can accept or donate a proton depending on the pH of the solution. As a result, the AH adjuvant has a pH-dependent surface charge. The point of zero charge or isoelectric point (iep) of AH is 11.4 giving AH a positive surface charge at neutral pH. Aluminum has a high affinity for phosphate, which can replace surface hydroxyls through ligand exchange. Aluminum has an even higher affinity for fluoride, moderate affinity for sulfate and low affinity for other anions such as chloride and nitrate.^16^ The adsorptive surface of AH adjuvant is therefore very dependent on the composition of the buffers in which it is used.

Aluminum phosphate adjuvant is prepared by mixing a solution of aluminum salt, usually AlCl_3_ or AlK(SO_4_)_2_, with a basic solution of trisodium phosphate, or by mixing aluminum salt with phosphate solution, followed by precipitation with sodium hydroxide. Substitution of hydroxyls for phosphate results in the formation of aluminum hydroxyphosphate, Al(OH)_x_(PO_4_)_y_, a nonstoichiometric compound in which the ratio of hydroxyls to phosphate depends on the precipitation conditions.^17^ AP is noncrystalline, because the incorporation of phosphate interferes with the crystallization process. Similar to AH, the surface hydroxyls of AP can accept or donate a proton which results in pH-dependent changes in the surface charge. Commercial AP preparations generally have a P:Al ratio of 1.1–1.15 and a point of zero charge of 4.6–5.6, which yields a negative surface charge at neutral pH.^18^ The point of zero charge can be decreased by replacing surface hydroxyls with anions such as phosphate, through ligand exchange. Addition of phosphate buffer to AH lowers the point of zero charge without changing the crystalline structure of the adjuvant.^19,20^ Dilution of AP adjuvant with a nonphosphate solution will reduce the number of phosphate groups at the surface and increase the point of zero charge.^21^ Hence the adsorptive surface and capacity of AP are also dependent on the buffer conditions and composition in which it is used. Amorphous aluminum hydroxyphosphate sulfate (AAHS) is similar to AP, but with a higher ratio of hydroxyls to phosphate than commercial AP. It contains residual sulfate because alum [AlK(SO_4_)_2_] was used as the source material instead of aluminum chloride.^22^ The AAHS adjuvant has a P:Al ratio of 0.3 and a point of zero charge of about 7.0.^22^

Another aluminum-containing adjuvant that is commonly used in preclinical experimental studies is Imject^TM^ Alum (ThermoFisher Scientific). However, this adjuvant is composed of amorphous aluminum hydroxycarbonate and crystalline magnesium hydroxide.^23^ In a direct comparison, the immune response induced by a vaccine formulated with AH was significantly stronger than that by a vaccine containing Imject^TM^ Alum.^24^ Because it has a different composition from AH and AP in licensed vaccines, Imject^TM^ Alum should not be used when the goal of the experiments is to formulate vaccines for preclinical evaluation, or even to evaluate mechanisms of adsorption. Moreover, it should be avoided entirely if the objective of the studies is to determine the mechanism of action of aluminum-based adjuvants.

## Formulation of vaccines with aluminum adjuvants

### Adsorption of antigens to aluminum adjuvants

**Fig. 2:**
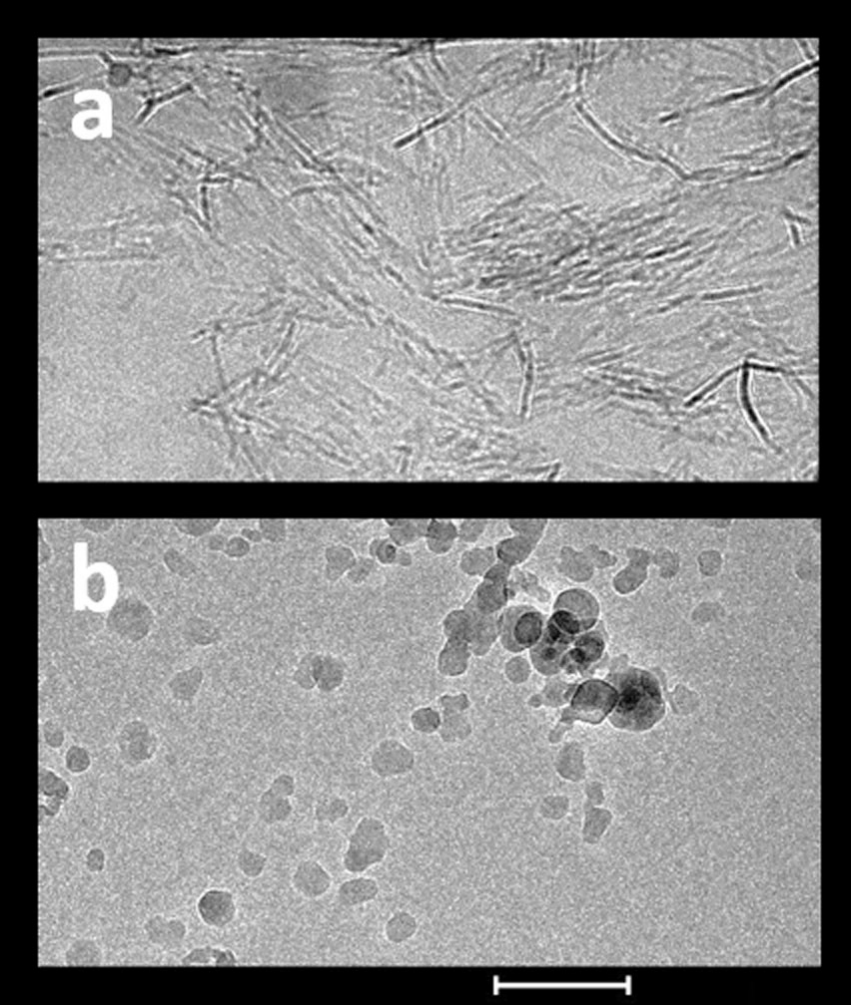
Structure of aluminum hydroxide adjuvant (**a**) and aluminum phosphate adjuvant (**b**). Images were obtained by cryo-electron microscopy. Scale bar = 200 nm

Aluminum adjuvants are composed of nanoscale primary particles (Fig. 2). The AH nanoparticles are elongate, approximately 4 × 2 × 10 nm in size, whereas the AP nanoparticles are plate-like with a diameter of approximately 50 nm.^25,26^ These nanoparticles form loosely connected porous aggregates that vary in size from 1 to about 20 µm depending on the adjuvant, the method used for measurement of particle size, and the experimental conditions.^27–30^ Exposure to shear forces and ultrasonication decreased the size of the aggregate adjuvant particles,^28,29^ whereas suspension of adjuvants in saline increased aggregation and the size of the aggregates.^30,31^ The ability of the aggregates to dissociate and reaggregate upon mixing contributes to the even distribution of adsorbed antigens in vaccine formulations.^32^ The primary nanoparticles that make up the aggregates provide a very large surface, estimated at 514 m^2^/g for AH (Rehydragel HPA), when measured by water adsorption using gravimetric infrared spectroscopy.^26^ A much smaller surface area was reported for Alhydrogel (350 m^2^/g) and Rehydragel LV (300 m^2^/g),^31^ but this was determined by nitrogen adsorption which significantly underestimates the surface area as a result of dehydration of the samples and collapse of the aggregates.^26^ During storage at room temperature, aluminum adjuvants become more ordered because of deprotonation and dehydration. This “aging” process reduces the surface area and resulted in a modest reduction of adsorptive capacity over a period of 15 months.^33^

Adsorption of antigens to aluminum adjuvants may contribute directly to the immune-enhancing effect of aluminum adjuvants as discussed below. In addition, adsorption to the adjuvant may prevent adsorption of antigens to the wall of the vial or syringe, thus ensuring injection of the full dose of antigen. Antigen adsorption to adjuvants occurs via hydrophobic, electrostatic, and ligand exchange mechanisms, among others. Purified or chemically synthesized antigens are large complex structures composed of proteins made up of a diverse array of 20 amino acids, sometimes conjugated with oligo- or polysaccharide chains and lipids. This diversity makes it difficult to predict the adsorptive behavior of antigens on aluminum adjuvants, as is also the case for the interaction of proteins with other solid surfaces.^34^ Proteins tend to adsorb to solid surfaces and adsorption is generally highest when the pH approaches the iep of the protein.^34,35^ Although most hydrophobic residues are buried inside globular proteins, proteins have hydrophobic residues at the surface that can interact with solid interfaces. The size and distribution of these hydrophobic patches determine the strength of the hydrophobic interactions.^36^ In addition, adsorption of protein antigens may result in partial unfolding, resulting in exposure of additional hydrophobic residues, which subsequently interact with the adjuvant surface.^34,35^ This can contribute to the increased strength of adsorption upon aging of certain antigen−adjuvant mixtures.^37^

Electrostatic interactions occur when the antigen and adjuvant have opposite charges and represent a major mechanism for adsorption of antigens to aluminum adjuvants. At neutral pH, AH has a positive surface charge and AP a negative surface charge. The iep of protein antigens can be used as a starting point to determine if it is likely to undergo electrostatic adsorption to either AH or AP.^38^ However, it is important to recognize that the iep represents the net of all charged groups of a protein and that the distribution of the charges will influence the interaction with the adjuvant particles.^39^ An extreme case from the literature comprises a fusion protein composed of two peptides, one with an iep of 10 and a second peptide with an iep of 5.5. Although the average iep was 9.3, it adsorbed equally well to both AP and AH presumably with different orientations.^40^

The strongest interaction between aluminum adjuvants and antigens is driven by the mechanism of ligand exchange. As mentioned above, aluminum has a high affinity for phosphate, and phosphate can exchange for hydroxyl groups at the surface of aluminum adjuvants.^19^ The presence and exposure of terminal phosphate groups in some antigens allows binding through ligand exchange. Binding of such antigens to aluminum adjuvants via ligand exchange can even overcome an electrostatic repulsion. Ligand exchange occurs with both types of aluminum adjuvants, but it is stronger in AH because this adjuvant has more surface hydroxyls available than AP. Ligand exchange may be involved in the binding of hepatitis B surface antigen (HBsAg) virus-like particles, possibly through the creation of terminal phosphate groups by limited hydrolysis of phospholipids.^41,42^ Terminal phosphate (PO_4_) or phosphonate (CH_2_-PO_3_) groups can also be added to protein antigens through conjugation with phosphoserine or with specifically designed linkers with one or more terminal phosphonate groups.^43,44^ Conjugation of lysozyme with two phosphonate linkers allowed binding of lysozyme to AH in spite of the fact that both compounds carried a positive charge.^44^ The availability of surface hydroxyl groups for ligand exchange can be determined by measuring the surface phosphophilicity.^45^ This assay uses a chromogenic or fluorogenic compound with a terminal phosphate group. Adsorption through ligand exchange results in hydrolysis and release of a colored or fluorescent product.

The degree of adsorption of antigens is usually calculated by determining the amount of protein in the supernatant following centrifugation of the antigen−adjuvant mixture, after short-term incubation. The amount of protein in the supernatant is simply subtracted from the amount that was initially added, to determine the amount adsorbed. By adding different amounts of antigen while keeping the adjuvant concentration constant, the adsorptive capacity of the adjuvant for the particular antigen can be determined by constructing an adsorption isotherm. This process is rather laborious, but can be readily automated using high-throughput liquid handling systems.^46–48^ The adsorption of protein antigens to aluminum adjuvants has been typically analyzed according to the Langmuir adsorption model, which allows calculation of several adsorptive parameters including the adsorptive coefficient, a correlate of the strength of the antigen−adjuvant interaction.^38,49^ However, although the adsorption isotherm can resemble the Langmuir model, the adsorption of proteins to solid surfaces does not meet the premises on which the Langmuir model is based.^35,50^ An alternative approach to determine the adsorptive strength is to assess the degree of desorption upon exposure to serum or lymph fluid, or to undertake studies in higher ionic strength buffers to compete off the antigens. However, evaluation in the presence of serum or lymph requires antigen-specific assays to determine the concentration of antigens in these complex fluids. Desorption can result from exposure to anions that undergo ligand exchange with surface hydroxyls on the adjuvant surface, or competitive displacement by other proteins such as fibrinogen.^44,51,52^ A quantitative desorption assay was developed for HBsAg based on the high affinity of aluminum for fluoride. The fluoride anion has a higher affinity for aluminum than phosphate and can desorb phosphorylated antigens such as HBsAg. The degree of desorption was inversely related to the adsorptive strength.^42^ An alternative method is to determine the effect of adsorption of antigens on the adsorption and hydrolysis of a fluorogenic compound. The decrease of hydrolysis (termed relative surface phosphophilicity) was inversely correlated with the affinity of the antigens.^47^ The adsorptive strength is an important parameter because too tight adsorption can interfere with the antibody response, as illustrated by the model antigen alpha-casein, which has eight phosphate groups available for ligand exchange, resulting in strong adsorption to AH.^53^ The strongly adsorbed casein did not induce T-cell activation possibly because adsorption interfered with antigen processing. Reduction of the adsorptive strength by pretreatment of AH with phosphate buffer allowed T-cell activation and resulted in a stronger antibody response.^53^ Similarly, the antibody response to HBsAg was enhanced by pretreatment of AH with phosphate buffer compared with AH alone.^41^ The adsorptive strength may also affect the degree of structural changes following adsorption and, hence, the integrity of conformational epitopes. The effect of adsorptive strength on the breadth and magnitude of the immune response is an area that requires further investigation, particularly with novel antigens.

The amount of protein adsorbed to aluminum adjuvants can also be determined directly. The *o*-phthalaldehyde (OPA) assay measures a fluorescent signal created when OPA reacts with terminal amine groups and has been used to determine the amount of adsorbed protein on AH.^54^ Limitations of the assay include its sensitivity, because it can only be used for protein concentrations greater than 25 µg/ml, and that it is unsuitable for some formulations, due to interference from excipients containing amine groups (such as Tris buffer). An alternative method is to measure total nitrogen content by chemiluminescence,^55^ although this technique is also subject to interference from excipients containing amine groups. In addition, the specific adsorption of antigens can be determined with panels of monoclonal antibodies by either plate-based fluorescence or flow cytometry.^56–59^ These assays require well-characterized antigen-specific antibodies, but have the advantage that the adsorption of specific antigen can be determined in combination vaccines that contain multiple antigens. In addition, such studies can provide information about the structural stability of the adsorbed antigens. One should be careful to avoid or control for direct adsorption of the antibodies to the aluminum adjuvant in the development of such assays. We have also described a Luminex®-based approach in which the degree of adsorption of several antigens can be evaluated simultaneously in a complex combination vaccine.^60^ The use of antibodies to evaluate antigenic integrity in adsorbed vaccines also offers the opportunity to develop in vitro potency assays.

Besides characterization of antigens adsorbed onto aluminum salts, it may also be important to understand the effects of antigens and other vaccine components on the physicochemical properties of the aluminum salt itself. Such changes could impact the reproducibility of experiments and product performance, if not well understood and controlled. Various characterization techniques are useful to elucidate the physicochemical properties of aluminum salts. Due to the heterogeneous nature of aluminum salts, particle size analysis is best approached using a combination of complementary techniques rather than relying on a single technique. For instance, many particle aggregates in AH and AP may be too large in size to be accurately assessed by dynamic light scattering. In this regard, laser diffraction particle sizing and optical and/or electron microscopy may be useful. Other relevant characterization techniques include X-ray diffraction for assessing the crystalline structure of aluminum formulations, microelectrophoresis for monitoring changes in zeta potential, and laser scattering optical profiling for quantifying the sedimentation rate.

Furthermore, it is important to note that processing of aluminum salts (e.g. for fill/finish of a vaccine product) can also introduce challenges. For instance, due to relatively rapid sedimentation rates, care must be taken to ensure that fill/finish processes are able to accurately control the amount of aluminum adjuvant in each vial. Aluminum ion content can be assessed by elemental analysis (e.g. inductively coupled plasma atomic emission spectroscopy).

### Effect of excipients on adsorption

As discussed above, phosphate has a high affinity for aluminum and can replace surface hydroxyls in AH and AP by ligand exchange. This lowers the surface charge of aluminum adjuvants and reduces the number of surface hydroxyl groups available for ligand exchange with phosphorylated antigens. Phosphate buffer should therefore be avoided in the formulation of vaccines with aluminum adjuvants, unless there is a specific rationale for its use. Other anions of commonly used buffers that can affect adsorption include borate, citrate, carbonate, and succinate.^61–64^ Buffers that do not appear to affect the surface characteristics of aluminum adjuvants nor the adsorption of antigens include histidine, MOPS, and Tris buffers. Vaccines should be isotonic to reduce pain upon injection. Isotonicity can be obtained by formulation with 150 mM NaCl, but NaCl can reduce the adsorption of electrostatically adsorbed antigens.^48,65^ This has been attributed to shielding of electric charges resulting in decreased electrostatic interactions. In addition, increased aggregation of primary AH particles results in a decrease of the adsorptive surface area.^31^

Surfactants are frequently added to vaccine formulations to increase antigen stability. In a study of the effect of different surfactants on adsorbed antigens, anionic and cationic surfactants caused desorption. In contrast, Triton® X-100, a nonionic surfactant, did not change the surface charge of AH and had no effect on adsorption.^66^ Although it is likely that other nonionic surfactants such as polysorbate 20 and polysorbate 80 also do not affect the adsorption of antigens on AH and AP, this needs to be further evaluated. Stabilizers may be added to protein antigens to enhance their thermal and pH-dependent stability (see below). Stabilizers such as sorbitol and glycerol can affect the adsorptive capacity of AH for proteins, but they do not appear to affect adsorption at the low concentrations that are typically used in vaccines.^67^ However, the effect of stabilizers on the adsorptive strength has not been thoroughly determined.

### Effect of adsorption on antigen stability

As proteins adsorb to solid surfaces such as aluminum adjuvant particles, they try to maximize the surface interactions by changing their conformation.^34,35^ This is a slow process that can increase the adsorptive strength over time. The structural changes can affect conformational epitopes and the stability of the proteins. The extent to which these changes occurs depends on the structure of the protein itself (“hard” vs. “soft” proteins), the tightness of binding to the surface, and the pH, among other factors.^68^ Proteins can undergo several chemical degradative processes, including deamidation of asparagine and glutamine residues, and hydrolysis or oxidation, which are pH-dependent.^68^ The positive surface charge of AH attracts negatively charged ions, forming the Stern layer in the immediate vicinity of the particle. The attraction of hydroxyl ions increases the pH of this layer by 1−2 units compared with the bulk pH of the vaccine formulation. Similarly, the pH of the Stern layer surrounding AP adjuvant particles will be lower.^69^ Thus, antigens that are adsorbed to aluminum adjuvants are exposed to a different pH than antigens that are present in solution, and this can accelerate deamidation and oxidation.^70^ The elevated microenvironment pH may also account for the hydrolysis of phosphodiester bonds in *Haemophilus influenzae* type b (Hib) capsular polysaccharide-protein conjugates following adsorption to AH^71^ since the hydrolysis is accelerated under alkaline conditions.^72^

In the process of vaccine development, it is important to characterize the stability of the antigens in solution, and to evaluate the effect of excipients. In a comprehensive approach, antigens are exposed to various conditions including variations in temperature, pH, buffers and potential stabilizers for various lengths of time. The effect on protein structure and aggregation is then determined by a panel of biophysical methods, potentially including circular dichroism, Fourier transform infrared (FTIR) spectroscopy, intrinsic and extrinsic fluorescence spectroscopy, and light scattering. The use of complementary methods increases confidence in the observations of protein structure. The data generated from these experiments can be analyzed by constructing empirical phase diagrams to identify optimal conditions for the stability of antigens.^73^ The outcome of these experiments guides the formulation with aluminum adjuvants, including the choice of adjuvants, the type and pH of the buffer, and the inclusion of stabilizers. The effect of formulation with adjuvant on antigen stability should be evaluated by a similar panel of orthogonal methods that were used to evaluate the antigens in solution. It has been demonstrated that the selection of stabilizers based on the stability of proteins in solution generally predicts well their effectiveness with adsorbed proteins.^67^ Adsorption of antigens to aluminum adjuvants can have no effect on their stability,^74–76^ decrease the stability,^67,76–80^ or enhance the stability.^76^ It has been suggested that decreased stability of adsorbed antigens might contribute to the immune enhancement effect of aluminum adjuvants.^77^ Aging of adsorbed diphtheria toxoid vaccines causes an increase in adsorptive strength, presumably reflecting increased structural changes, and this is associated with an enhanced immune response.^12^ On the other hand, changes in the conformation of antigens may cause loss of critical B-cell epitopes. Storage of an anthrax vaccine composed of recombinant protective antigen adsorbed to AH led to structural changes and loss of the ability to induce neutralizing antibodies.^81^ The effect of adsorption on the accessibility and integrity of B-cell epitopes can be readily probed with a panel of monoclonal antibodies using flow cytometry, solid phase or surface plasmon resonance assays.^56–59,82^ Antigens adsorbed by relatively weak interactions readily desorb upon exposure to interstitial fluid following injection of a vaccine.^83,84^ Some antigens that have undergone partial unfolding following adsorption can refold following desorption. From these observations, it should be clear to conclude that further antigen-specific evaluations are necessary to assess the relationship between adsorption-induced changes in stability and the quality of the immune response induced.

### Effect of adjuvant dose

The amount of aluminum adjuvant in vaccines is typically expressed as the concentration of elemental aluminum per dose. One mg of Al^3+^ corresponds with 2.2 mg AH and about 4.5 mg AP.^85^ The maximum amount of aluminum per dose in human vaccines in the United States is 0.85 mg Al^3+^ if determined by assay and 1.14 mg if determined by calculations (US Code of Federal Regulations, 21CFR610.15). The amount may be increased to 1.25 mg if supported by data that this dose is safe and necessary to induce the desired effect. Licensed human vaccines typically contain between 0.2 and 0.8 mg of Al^3+^^86^ (Table 1). Because of the large adsorptive capacity of aluminum adjuvants, only a small dose is necessary to adsorb vaccine antigens. However, a larger dose than required for maximal adsorption is sometimes necessary to achieve an optimal immune response.^87^ Preclinical studies should be conducted to determine the minimum dose of aluminum adjuvant that induces a maximal immune response. For preclinical studies in rodents, the vaccine formulation intended for human use should not be diluted with saline or buffer as this can affect the adsorption of vaccine antigens. Instead, a fraction of the human dose, typically one-fifth or one-tenth (0.1–0.05 ml) for mice, should be used.^88^

**Table 1:** Type of adjuvant and aluminum content of aluminum-adjuvanted vaccines licensed for use in the United States (Information accessed on FDA website on May 24, 2018 (https://www.fda.gov/BiologicsBloodVaccines/Vaccines/ApprovedProducts/ucm093833.htm))

Vaccine	Tradename	Manufacturer	Adjuvant	Dose (Al^3+^)
Anthrax	BioThrax®	Emergent Biosolutions	AH	1.2 mg
Diphtheria and Tetanus Toxoids Adsorbed		Sanofi-Pasteur	AP	0.33 mg
DTaP	Daptacel®	Sanofi-Pasteur	AP	0.33 mg
	Infanrix®	GSK	AH	≤0.625 mg
DTaP, Hepatitis B, polio	Pediarix®	GSK	AH	≤0.85 mg
DTaP, polio	Kinrix®	GSK	AH	≤0.6 mg
	Quadracel®	Sanofi-Pasteur	AP	0.33 mg
DTaP, polio, Hib	Pentacel®	Sanofi-Pasteur	AP	0.33 mg
Hib	PedVaxHIB®	Merck	AAHS	0.225 mg
Hepatitis A	Havrix®	GSK	AH	0.5 mg (adult)
				0.25 mg (pediatric)
	VAQTA®	Merck	AAHS	0.45 mg (adult)
				0.225 mg (pediatric)
Hepatitis A and Hepatitis B	Twinrix®	GSK	AH and AP	0.45 mg
Hepatitis B	Engerix	GSK	AH	0.5 mg (adult)
				0.25 mg (pediatric)
	Recombivax HB®	Merck	AAHS	0.5 mg (adult)
				0.25 mg (pediatric)
Human papilloma virus	Cervarix®	GSK	AH	0.5 mg (plus 50 µg MPLA)
	Gardasil®	Merck	AAHS	0.225 mg
	Gardasil-9®	Merck	AAHS	0.5 mg
Meningococcus B	Bexsero®	GSK	AH	0.519 mg
	Trumenba®	Pfizer	AP	0.25 mg
Pneumococcus	Prevnar 13®	Pfizer	AP	0.125 mg
Tetanus and Diphtheria Toxoids, Adsorbed	Tenivac®	MassBiologics	AP	≤0.53 mg
		Sanofi-Pasteur	AP	0.33 mg
TdaP	Adacel®	Sanofi-Pasteur	AP	0.33 mg
	Boostrix®	GSK	AH	≤0.39 mg

*DTaP* diphtheria toxoid, tetanus toxoid, acellular pertussis, *Hib* *Haemophilus influenzae* B, *TdaP* tetanus toxoid, reduced diphtheria toxoid, acellular pertussis, *AH* aluminum hydroxide adjuvant, *AP* aluminum phosphate adjuvant, *AAHS* amorphous aluminum hydroxyphosphate sulfate

## Mechanism of action of aluminum adjuvants

Although aluminum adjuvants have been used for over 70 years in human vaccines, the mechanism by which they enhance the immune response remains not fully understood. This probably reflects the fact that several mechanisms are operating simultaneously, rather than a lack of awareness of an individual mechanism. Studies aimed at elucidating the mechanisms by which aluminum adjuvants enhance the immune response have recently been reviewed in detail.^4,9,89^ Here, we will provide a brief interpretive summary with updates from recent studies. A satisfactory explanation of the mechanisms that underlie the immune-enhancing effect of aluminum adjuvants should take into account that adsorption of antigen, although not always, usually enhances the immune response. Adsorption is probably important for three reasons: (1) it makes soluble antigens particulate, which enhances uptake through phagocytosis by dendritic cells;^27,90,91^ (2) it targets antigen to antigen-presenting cells, while enhancing antigen-presentation, as indicated by increased expression of MHC II-peptide complexes and increased activation of CD4 T cells;^91–93^ and (3) it retains antigen at the injection site, allowing time for recruitment of antigen-presenting cells through the release of cytokines and the induction of a local inflammatory reaction.^94–96^ It has been suggested that a short-term depot is not necessary for the effect of aluminum adjuvants.^97^ This was based on the intradermal injection of protein adsorbed to aluminum adjuvant in the ear of mice (the ear of mice does not have muscle or subcutaneous tissue) followed by surgical resection of the ear at different time points. Removal of the ear as early as 2 h after injection had no effect on the immune response. However, although the study was a sincere attempt to examine the need for a depot effect, the authors could not exclude the possibility that tissue damage from the resection of the ear resulted in sufficient inflammatory signals to enhance the immune response. An early study showed that removal of the injection site after subcutaneous injection of an aluminum-adjuvanted vaccine within 4 days after injection interfered with the immune response.^98^ Thus, it seems likely that a short-term depot is generally needed for the immunostimulatory effect of aluminum adjuvants.

Injection of aluminum-adjuvanted vaccines induces a limited amount of necrosis of tissue cells at the site of injection, which may lead to the limited release of some “danger-associated” molecular patterns, including DNA,^99,100^ uric acid,^101^ ATP,^102^ heat shock protein-70,^103^ IL-1α^104,105^ and IL-33,^106,107^ which are molecules that recruit and activate inflammatory cells. The multitude of released factors suggest significant redundancy and explains why inhibition or deletion of one of these molecules typically has limited or no effect on immune enhancement. The relative role of each factor likely depends on experimental variables such as the route of injection, dose, volume, and genetic background of the mouse strain used. The recruitment of inflammatory cells into the injected skeletal muscle follows an organized kinetic pattern, with an early and most abundant accumulation of neutrophils, followed by monocytes and macrophages and then eosinophils.^95,108^ Of note, this pattern is different following intraperitoneal injection, with more rapid accumulation of eosinophils and depletion of residential peritoneal macrophages,^101,105^ and in secondary responses in which eosinophil accumulation occurs earlier and is more prominent.^95^ Dying neutrophils at the injection site release DNA and form neutrophil extracellular traps (NETs).^109^ Lack of formation of NETs in mice deficient in peptidylarginine deiminase 4 inhibited the IgG1 response to AH-adsorbed ovalbumin, suggesting a role for neutrophils in the immunostimulatory activity of aluminum adjuvants. However, other studies showed that depletion of neutrophils did not affect the IgG1 or IgG2a responses to AH-adsorbed ovalbumin,^95^ and enhanced the antibody response to lysozyme mixed with AH.^110^

Dendritic cells play a critical role in the immune-enhancing effect of aluminum adjuvants as depletion of these cells impairs the immune response.^101^ Studies in non-human primates showed that the activation of antigen-specific T cells is restricted to the draining lymph nodes following injection of vaccines formulated with different adjuvants, including aluminum adjuvants.^111^ Aluminum adjuvants increase the transport of antigens via migratory dendritic cells from the injection site to the draining lymph node.^96,111,112^ Aluminum adjuvants induce the differentiation of monocytes into dendritic cells^92,113,114^ and activate dendritic cells, resulting in secretion of IL-1β and more efficient antigen presentation.^91,93,115^ The secretion of IL-1β requires processing of pro-IL-1β into a bioactive form of IL-1β. Aluminum adjuvants activate the NLRP3 inflammasome which results in cleavage of pro-IL-1β into IL-1β by caspase-1.^107,116–119^ However, deletion of MyD88, which is required for signaling through the IL-1 receptor, does not impair the antibody response to aluminum-adjuvanted vaccines, suggesting a redundant role of IL-1β in the immune-enhancing effect of aluminum adjuvants.^120,121^ Aluminum adjuvants can activate other signaling pathways in dendritic cells, including phosphoinositide 3-kinase and calcineurin-NFAT.^122–124^ Both pathways may be initiated by the binding of aluminum adjuvants to cell membrane lipids and were dependent on Syk. Activation of NFAT required LPS priming of dendritic cells and led to the secretion of IL-2. Inhibition of IL-2 expression specifically in dendritic cells reduced the antibody response to aluminum-adjuvanted vaccines.^123^ However, the mouse immunization experiments in this report were carried out with Imject^TM^ Alum, and the role of NFAT and DC-secreted IL-2 in the immune response to vaccines formulated with AH and AP adjuvants remains to be determined.

Aluminum hydroxide adjuvant also activates the complement cascade.^125,126^ The activation involves predominantly the alternative pathway with minor contributions from the classical and lectin pathways, and results in the release of C3a and C5a, as well as formation of the membrane attack complex (MAC).^126^ C3a and C5a may contribute to the recruitment of inflammatory cells to the site of injection, whereas MAC can be inserted into the membrane of macrophages and induce activation of the NLRP3 inflammasome.^127^ Complement activation plays a critical role in the humoral immune response^128^ and activation of complement may be an important, but underappreciated, mechanism underlying the immune-enhancing effect of aluminum adjuvants.

## Aluminum-containing combination adjuvants

The combination of immunostimulatory molecules that target different mechanisms to activate the immune response and aluminum can have synergistic effects that may result in a more effective or longer lasting immune response, and allow for a smaller amount of antigen in the vaccine (dose sparing). Aluminum adjuvants induce only weak Th1 and Th17 responses, which may be necessary for the induction of protective immunity against certain infectious diseases, such as malaria and tuberculosis. Adsorption of immunostimulatory molecules to aluminum adjuvants limits the systemic distribution of the molecules, which reduces the risk of systemic side-effects, and enhances the targeting of such molecules and co-adsorbed antigens to antigen-presenting cells recruited to the injection site.^129,130^ Ligands for pattern recognition receptors, in particular the Toll-like receptors (TLRs), are excellent candidates for combination adjuvants (Table 2) as they are localized at the cell membrane or in endosomal compartments and signal via MyD88 and TRIF pathways, that are complementary to aluminum adjuvant-activated cell signaling pathways. The AS04 adjuvant, comprised of aluminum adjuvants with the TLR4 agonist monophosphoryl lipid A, was the first combination adjuvant to be approved for use in licensed vaccines against human papilloma virus and hepatitis B.^131^

**Table 2:** Examples of aluminum-containing combination adjuvants in vaccines licensed for use or under clinical development (not comprehensive)

Molecular adjuvant (receptor)	Aluminum adjuvant	Indication(s)	Development stage
MPL® (TLR4)	AH or AP	Human papilloma virus, Hepatitis B	Licensed (Cervarix®, Fendrix®)
CpG (TLR9)	AH	Malaria, Hookworm, Nicotine	Phase 2
GLA (TLR4)	AH	Hookworm	Phase 1
LHD153R (TLR7)	AH	Meningococcus	Phase 1
QS-21	AH	HIV	Phase 1
IL-12	AH	Leishmaniasis	Phase 1

*AH* aluminum hydroxide adjuvant, *AP* aluminum phosphate adjuvant

The formulation of aluminum adjuvants with other immunostimulatory molecules is subject to the same considerations as outlined above for vaccine antigens. The TLR4 ligands LPS and MPL are negatively charged and have one or two terminal phosphate groups, which allow for adsorption to AH by electrostatic interactions and ligand exchange.^132,133^ Polynucleotides such as poly (I:C), a TLR3 ligand, and CpG oligonucleotides (ODN), which bind TLR9, are negatively charged and adsorb strongly to AH, but not to AP.^129^ The adsorption of CpG ODN to AH was reduced in the presence of phosphate buffer.^129^ Adsorption of CpG ODNs was required to obtain an increased antibody response in comparison with AH alone. When the dose of CpG ODNs was increased to exceed the adsorptive capacity of AH, the immune response was reduced.^134^ The adsorption of small molecules such as TLR7 ligands that lack a terminal phosphate group or a strong charge requires additional procedures. Insoluble lipophilic molecules can be incorporated into lipid nanosuspensions comprising specifically selected charged phospholipids.^135^ Negatively charged nanoparticles adsorbed to AH, but not to AP. The combination adjuvant enhanced antigen-specific antibody production and induced IFN-γ-secreting CD4 T cells consistent with a Th1 response.^135^ In an alternative approach, a small molecule TLR7 agonist was synthesized with a linker molecule that contained a terminal phosphonate group.^130,136,137^ This allowed the direct binding of the TLR7 ligand to AH via ligand exchange as previously demonstrated for protein antigens.^44^ The physicochemical properties of the AH/TLR7 combination adjuvant were thoroughly characterized by a suite of techniques including ultraperformance liquid chromatography, confocal microscopy, flow cytometry, zeta potential, phosphophilicity assay, Raman spectroscopy, nuclear magnetic resonance, and mass spectroscopy.^137^ The biological activity of the adjuvant included enhanced antibody response and induction of both Th1 and Th17 CD4 T cells.^130,138^

The adsorption of TLR ligands may affect the surface charge of the aluminum adjuvants and the adsorptive capacity and strength for antigens. Indeed, adsorption of CpG ODNs reduced the adsorption of some, but not all antigens.^129^ Thus, the ratio of TLR ligands to aluminum adjuvants and order of mixing should be optimized to allow adequate adsorption of antigens and optimal stimulation of the immune response.

## Future directions of aluminum adjuvants

Besides active investigation of aluminum-based combination adjuvants and mechanisms of action, several research groups have reported attempts to develop well-defined aluminum nanoparticle adjuvants, with reduced particle size compared to the available commercial products.^139–142^ If such efforts are successful, aluminum nanoparticles with tunable particle size and even particle shape could potentially offer approaches to optimize biological activity (e.g. lymph node trafficking) and manufacturing/stability aspects (e.g. ability for terminal sterile filtration), beyond what is achievable with the traditional aluminum salts. Nevertheless, in most cases the novel adjuvant nanoparticles are not benchmarked to commercial aluminum salts, making it difficult to evaluate benefit and supporting our motivation in writing this review. Other interesting developments which could facilitate delivery of aluminum salt vaccines to resource poor areas include lyophilized thermostable formulations and excipient approaches that enable stability to freezing temperature excursions.^64,143^ Moreover, there are plenty of opportunities for the development of new and improved characterization tools, to attain a better understanding of the binding, release, and stability of aluminum-adsorbed antigens.

In this review we are not attempting to “reinvent” aluminum adjuvants, nor to suggest that they are more potent than has already been established through many years of experience. For example, we recently published a study in non-human primates that evaluated a number of alternative adjuvants, including AH, with a recombinant HIV env protein, in which the relative lack of potency of the aluminum-adjuvanted vaccine was clear.^144^ Nevertheless, we strongly believe that aluminum adjuvants remain a key benchmark and a “gold standard”, against which new adjuvants can and should be routinely evaluated. The safety and potency of aluminum adjuvants has been established in man, in combination with many vaccines, over decades, involving billions of doses, and the accumulated experience represents an important and substantive “body of evidence”. Although newer adjuvant approaches can and will be shown to be more potent than aluminum, the enhanced potency needs to be critically appraised, rather than simply touted as an unquestioned advantage. The level and type of immune response necessary for protective immunity for a particular vaccine is often unknown and needs to be determined in large clinical studies, but you only need enough, not more. A recent study compared the innate and adaptive immune response to vaccination of human volunteers with an HBsAg vaccine formulated with AH or several newer adjuvants.^145^ The authors found that the antibody response to HBsAg exceeded the minimum protective level with all formulations, but it reached a higher level in individuals vaccinated with the newer adjuvants than in those vaccinated with AH. This was, however, associated with increased activation of the innate immune response and local and systemic reactogenicity.^145^

We are proposing two key practical considerations for adjuvant research and development. One, that aluminum adjuvants continue to be a necessary benchmark in all studies, and two, that they need to be used appropriately, with some formulation optimization, to enable it to be a legitimate comparison. Our expectation is that the potency of aluminum adjuvants will continue to be exceeded by alternative approaches in preclinical and clinical studies, but there needs to be a vaccine-specific evaluation of how much enhancement is enough, in addition to how much reactogenicity is acceptable. In many programs, the development timelines, the manufacturing and component sourcing challenges, the accumulated data necessary on new approaches, and the regulatory challenges involved, will all be considerably reduced if an aluminum-based adjuvant is selected for product development. Particularly if it can be determined that an aluminum adjuvant can provide enough to accomplish what is needed. This will be particularly true as new generation antigens are developed, with key structural elements to render them inherently more potent as immunogens. Moreover, enthusiastic scientists promoting more novel approaches should not lose sight of the inherent value in using the preferred presentation modes for vaccine products in the market place, single vial liquids or prefilled syringes, which can be readily accomplished with traditional aluminum-adjuvanted vaccines.
